# Identification of a minimum number of genes to predict triple-negative breast cancer subgroups from gene expression profiles

**DOI:** 10.1186/s40246-022-00436-6

**Published:** 2022-12-20

**Authors:** Laila Akhouayri, Paola Ostano, Maurizia Mello-Grand, Ilaria Gregnanin, Francesca Crivelli, Sara Laurora, Daniele Liscia, Francesco Leone, Angela Santoro, Antonino Mulè, Donatella Guarino, Claudia Maggiore, Angela Carlino, Stefano Magno, Maria Scatolini, Alba Di Leone, Riccardo Masetti, Giovanna Chiorino

**Affiliations:** 1 Department of Biomedical Sciences, Genetics and Molecular Biology Laboratory, Faculty of Medicine and Pharmacy, Hassan II-Casablanca University, Casablanca, Morocco; 2 Department of Life Sciences and Systems Biology, University of Turin, Turin, Italy; 3 Cancer Genomics Lab, Fondazione Edo ed Elvo Tempia, Biella, Italy; 4 Clinical Research Division, “Degli Infermi” Hospital, Ponderano, BI Italy; 5 Molecular Oncology Lab, Fondazione Edo ed Elvo Tempia, Biella, Italy; 6 Pathology Department, “Degli Infermi” Hospital, Ponderano, BI Italy; 7 Oncology Department, “Degli Infermi” Hospital, Ponderano, BI Italy; 8 Fondazione Policlinico Universitario A. Gemelli IRCCS, Rome, Italy

**Keywords:** TNBC, Prediction, Personalised medicine, Data mining, Machine learning, Druggable targets, Gene network analysis

## Abstract

**Background:**

Triple-negative breast cancer (TNBC) is a very heterogeneous disease. Several gene expression and mutation profiling approaches were used to classify it, and all converged to the identification of distinct molecular subtypes, with some overlapping across different approaches. However, a standardised tool to routinely classify TNBC in the clinics and guide personalised treatment is lacking. We aimed at defining a specific gene signature for each of the six TNBC subtypes proposed by Lehman et al. in 2011 (basal-like 1 (BL1); basal-like 2 (BL2); mesenchymal (M); immunomodulatory (IM); mesenchymal stem-like (MSL); and luminal androgen receptor (LAR)), to be able to accurately predict them.

**Methods:**

Lehman’s TNBCtype subtyping tool was applied to RNA-sequencing data from 482 TNBC (GSE164458), and a minimal subtype-specific gene signature was defined by combining two class comparison techniques with seven attribute selection methods. Several machine learning algorithms for subtype prediction were used, and the best classifier was applied on microarray data from 72 Italian TNBC and on the TNBC subset of the BRCA-TCGA data set.

**Results:**

We identified two signatures with the 120 and 81 top up- and downregulated genes that define the six TNBC subtypes, with prediction accuracy ranging from 88.6 to 89.4%, and even improving after removal of the least important genes. Network analysis was used to identify highly interconnected genes within each subgroup. Two druggable matrix metalloproteinases were found in the BL1 and BL2 subsets, and several druggable targets were complementary to androgen receptor or aromatase in the LAR subset. Several secondary drug–target interactions were found among the upregulated genes in the M, IM and MSL subsets.

**Conclusions:**

Our study took full advantage of available TNBC data sets to stratify samples and genes into distinct subtypes, according to gene expression profiles. The development of a data mining approach to acquire a large amount of information from several data sets has allowed us to identify a well-determined minimal number of genes that may help in the recognition of TNBC subtypes. These genes, most of which have been previously found to be associated with breast cancer, have the potential to become novel diagnostic markers and/or therapeutic targets for specific TNBC subsets.

**Supplementary Information:**

The online version contains supplementary material available at 10.1186/s40246-022-00436-6.

## Background

Triple-negative breast cancer (TNBC) affects approximately 15% of women with mammary tumours. The so-called TNBC is an immunohistochemical definition corresponding to the absence of oestrogen (ER) and progesterone (PgR) receptors expression and of the human epidermal growth factor receptor 2 (HER2) amplification.

TNBC are large, high-grade carcinomas with a high Ki67 mitotic index and numerous nuclear atypia on anatomo-pathological examination. These cancers are often related to the basal subtype, introduced for the first time by Podo et al. and Perou et al. in their paramount work [1, 2], and have similarities with cancers developed on germline BRCA mutation. The basal-like subtype (BL) is characterised by basal cytokeratin gene overexpression and the absence of oestrogen, progesterone and HER2 coding genes expression. BRCA1/2 gene mutations are found in approximately 30% of cases [3]. TNBC is usually associated with a younger age at diagnosis, aggressive profile and high rates of p53 gene mutations, accompanied by strong immunohistochemically detected p53 [4]. They present a high risk of relapse, despite greater sensitivity to chemotherapy, and of metastatic recurrence in the first 3 years after diagnosis. They are not eligible for treatments targeting hormone receptors or HER2. However, in addition to chemotherapy, these cancers may benefit from new treatment options, depending on the tumour nature. Since 2005, the intensive development of high-throughput technologies to analyse gene mutation status and/or expression has increased the knowledge of the genomic and phenotypic profiles of TNBC [5].

First, several subcategories can be identified by analysing their morphology and some have either a particular prognosis, or a specific therapeutic response [6]. Second, high-tech throughput technologies, thanks to the analysis of thousands of genes, have begun to show TNBC molecular subclasses, exhibiting specific molecular abnormalities associated with response to treatment and/or to survival [7]. Thirdly, evidence has accumulated, showing that TNBC microenvironment, the cells and molecules present in the tumour stroma play a significant role in disease progression [8]. Thus, the characteristics of the microenvironment can serve as a new TNBC subclassification basis with a potential therapeutic impact [9].

In 2011, Lehman BD and colleagues [10] proposed a Web-based subtyping tool through which six TNBC subgroups were identified, based on high-throughput gene expression profiling of several hundreds of TNBC samples. Various expression abnormalities related to cell cycle regulatory genes, such as BRCA2 and DNA repair ones (TP53), were detected in the basal-like type 1 (BL1) subtype. The second basal-like subtype (BL2) was more associated with abnormal activation of other signalling pathways, such as EGFR, MET, cell migration, extracellular matrix–receptor interaction and differentiation. Contrariwise, the mesenchymal stem-like (MSL) subtype was more associated with underexpression of cell proliferation and overexpression of mesenchymal stem cell-related genes. The immunomodulatory (IM) subtype was mainly recognised by immune signal transduction pathways, such as those related to NK, B, dendritic and T cell gene expression. The mesenchymal (M) subtype, on the other hand, was enriched in cell migration-related signalling pathways as well as extracellular matrix–receptor interaction and differentiation pathways. The luminal androgen receptor (LAR) subtype was very different from all the others: although being  ER-negative, it expressed the androgen receptor (AR) and/or its downstream effectors, and was highly associated with hormonal-related signalling pathways, such as steroid synthesis and androgen/oestrogen metabolism.

Thereafter, Burstein supervised another study where copy number variations (CNV) analysis and genomic profiling techniques were employed to furthermore stratify TNBC, finding four different subtypes with distinct prognosis: LAR, MES (mesenchymal), BLIS (basal-like immunosuppressed) and BLIA (basal-like immune-activated) [11].

On the other hand, in a more recent study by Jézéquel et al., three distinct subtypes were highlighted by transcriptomic profiling techniques. The first is recognised by an apocrine molecular phenotype showing favourable prognosis, the other two groups had more basal properties: while one was more aggressive and coupled with an immunosuppressive phenotype, the third showed adaptive immune response [12].

Finally, another study developed by Liu et al. and based essentially on long-non-coding RNAs (lncRNAs) expression resulted in the development of the Fudan University Shanghai Classification System (FUSCC) with four subtypes: IM, LAR, MES, and BLIS, with upregulation of proliferative pathways and the worst overall survival in the latter [13].

However, the potential driving molecular events within each TNBC subtype, as well as their response to personalised treatment, remain seldom explored. Further insights into the underlying genomic alterations, as well as towards a standardised and easily applicable subclassification, are therefore needed. Under the perspective of integrating a molecular portrait into clinical practice and starting from Lehman's classification, we aimed at identifying a limited number of genes that can serve as a genetic signature for the prediction of the different TNBC subtypes.

## Materials and methods

### Data description

Two TNBC data sets were downloaded from public repositories. The first one was retrieved from the Gene Expression Omnibus (GEO) and refers to whole transcriptome RNA sequencing (RNA-seq) performed on pre-treatment research biopsies from the BrighTNess phase III study (AFT-04). This data set (GSE164458) consists of log-normalised RNA-seq expression values of clinical stages II to III tumours. It will be called GEO-TN [14].

The second one was retrieved from the Genomic Data Commons (GDC) Data Portal of the National Cancer Institute and refers to the cancer genome atlas (TCGA) project: only TNBC samples were selected, based on their ER-, PgR- and HER2-negative immunohistochemical status, for a total of 63 TNBC records out of 1093 invasive BC records. This data set contains log-normalised RNA-seq expression values and clinical data. It will be called TCGA-TN [15].

The third data set was uploaded to the public repository under the GEO accession number GSE206912 and refers to 72 TNBC from Italian patients surgically treated at the Hospital of Biella or at the Policlinico Gemelli in Rome, that underwent gene expression profiling at the Genomics Lab of Fondazione Edo ed Elvo Tempia, Biella (Italy). It will be called Italian-TN. Sample collection was approved by the Ethical Committees of Novara and Policlinico Gemelli (Prot. 861 CE 149/19 and Prot. 3559, respectively). After tumour area selection, total RNA was isolated from macrodissected sections using the Agilent Absolutely RNA FFPE Kit, reverse-transcribed to the corresponding cDNA and in vitro transcribed with the Sigma TransPlex Whole Transcriptome Amplification Kit; cDNA was amplified and labelled with the Agilent SureTag DNA Labeling Kit; hybridised by means of the Agilent Gene Expression Hybridization Kit on whole genome SurePrint G3 Human GE 8 × 60 K V3 microarrays containing probes for 26,803 coding RNAs and 30,606 lncRNAs; slides were washed using the Gene Expression Wash Buffer Kit and then scanned with the Agilent scanner version C. All protocols and kits were purchased from Agilent Technologies. After scanning, array image analysis was carried out using the Agilent Feature Extraction Software v12.1, and then raw expression data were processed by background subtraction (*normexp* function, with offset = 50) followed by between array quantile normalisation, using the *LIMMA* (LInear Models for Microarray Analysis) package in R software for Statistics v.4.1.0. This data set contains log-normalised intensities.

### TNBC-subtype prediction

Before subtype prediction, the *dplyr* package on R was used to remove non-expressed genes in all the samples (with null expression values). Pre-processed data from the GEO-TN, TCGA-TN and Italian-TN data sets were then uploaded in the TNBCtype online tool [16], which first investigates the presence of any hormone receptor-positive sample and removes it. Then, it calculates the Spearman correlation (and its significance) between each sample and the six centroids of the TNBC subtypes previously determined and assigns samples to the most correlated subtype. UNS is assigned to unstable samples, with very low and not statistically significant correlation with any subtype. UNS samples were excluded from downstream analyses.

### Gene signature determination

This step is based on the calculation of differentially expressed genes (DEGs) specific to each TNBC subtype, in contrast to the others. Two different methods were selected to have the best DEGs pick. The first one was class comparison using the *LIMMA* package in R, where differentially expressed genes between each predicted TNBC subgroup and the remaining samples were obtained by combining a modified t test with empirical Bayes modelling, in order to moderate the standard errors of the estimated log-fold changes. The detection of differential gene expression was done by applying a cut-off to the Benjamini and Hochberg adjusted *p* values (< 0.01). The second method used was the mean difference based on Mann–Whitney *U* (MWU) test, using the same method to adjust p values for multiple test comparisons. The detection of differential gene expression was done by applying a cut-off to the adjusted *p* values (< 0.01) and to the difference in median expression between subgroups (LogFC ≥ 1 and < − 1) for up- and downregulated genes, respectively. Both methods outcomes were combined by the *merge* function from the *dplyr* package in R for further analysis.

### TNBC subtypes network analysis and identification of druggable targets

Functional analysis of differentially expressed genes was performed using the Web-based tool MetaCore™ version 22.1 software suite (Clarivate Analytics, Philadelphia, PA, USA). Gene network analysis was carried out using Dijkstra's Shortest Path algorithm to find the shortest path between gene (or gene product) pairs, in each direction, allowing for one step (direct interactions) or two steps (one additional network object inserted as intermediary interaction).

As for the druggable targets analysis, we looked for therapeutic drug–target interactions (experimentally validated) and secondary drug–target interactions that are just predicted based on similarities in the structures.

### Subtype prediction according to the genetic signature

This step was assessed by *Weka v3.9.3 software for data mining*. The “subtype membership” was considered as the variable of interest, while all the other attributes (selected genes) were used as predictive variables. Relevant machine learning algorithms were therefore selected to compare and evaluate the model performance. The following models were used: naive Bayes (NB), logistic regression (LR), decision tree (DT), random forest (RF), support vector machine (SVM), *K*-nearest neighbours (KNN) classifier, and multilayer perceptron (MP).

The analysis included an automatic feature engineering, which is based on a *k*-fold cross-validation, where the original sample is partitioned into k subsets. The model was trained on all but one subsets (*k* − 1) and then evaluated on the subset that was not used for training. This cross-validation process was systematically repeated k times (the folds), where each of the k subsets was used exactly once as validation data (and excluded from training) each time. The *k*-fold results were then averaged (or otherwise combined) to produce a single final estimate. *K* was set = 10.

### Prediction evaluation metrics

Each prediction model was evaluated by ten different metrics, such as true positive (TP) rate, false positive (FP) rate, accuracy, Cohen’s kappa, precision, recall, F-measure, Matthews correlation coefficient (MCC), receiver operating characteristic curve (ROC) area and precision–recall curve (PRC) area.

### Best attribute selection

This step was useful to choose a small subset of features (genes) that was sufficient enough to effectively classify the target class (TNBC subtype), by reducing computational cost and improving accuracy. Accordingly, the prediction quality of each gene of the training data set was evaluated and the genes that provided less value (voted by the majority rule of different attribute selection algorithms) were discarded. Seven different attribute selection algorithms were used by *Weka software*: Pearson's correlation; information gain; symmetrical uncertainty; Cf subset; gain ratio; relief F; and one R.

Their central hypothesis is that the important attribute sets are strongly correlated with the target class, and uncorrelated attributes are less important. Further, strong correlation among attribute pairs makes only one of them important and the other one can be removed. If two or more attributes have the same importance to the target class values, only one of them is considered.

The final attribute selection methods list gathers the results of the ranking of all the attributes from the most to the least important. Only genes that were ranked as unimportant by at least four out of seven algorithms were then highlighted as the least important attributes.

## Results

### TNBC subtypes prediction and gene signature determination

**Fig. 1:**
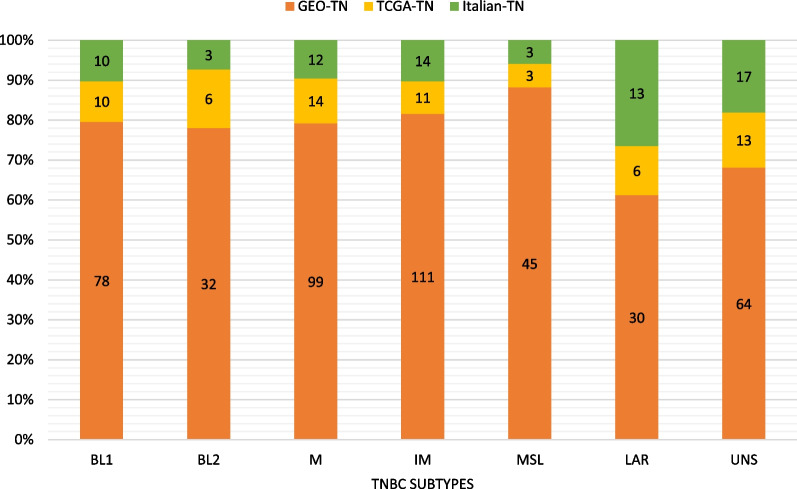
Predicted subtype counts in GEO-TN, TCGA-TN and Italian-TN data sets by TNBCtype tool, using whole transcriptomic data

All the three TNBC data sets were subtyped using the TNBCtype online tool. For the GEO-TN data set, there were 23 ER + detected and 64 UNS predicted samples, which were discarded. Accordingly, the final number of samples obtained was 395. This data set is by far the largest and was used as a training set. The TCGA-TN data set initially consisted of 63 records from which 13 unstable ones were discarded, resulting in 50 TNBC samples. Seventeen samples were predicted as UNS and were therefore automatically eliminated from the Italian-TN data set, which resulted in a final number of 55 samples. The two latter were used as validation sets. Subtyping results for the three data sets are detailed in Fig. 1. The IM and M subtypes were the most prevalent, while BL2 and LAR were the least frequent, which can give us an idea about the subgroup imbalance.

The two tests used to determine differentially expressed genes converged on the most significant genes within each subgroup in contrast to the others. Subsequently, two gene lists were generated, the first with the 120 most upregulated (Additional file 1: Table S1) and the second with the 81 most downregulated genes (Additional file 1: Table S2).

**Table 1:** Comparative overview of seven prediction algorithms according to the 120 upregulated genes

	TP rate	FP rate	Accuracy %	Mean absolute error	Kappa	Precision	Recall	F-measure	MCC	ROC area	PRC area
Naive Bayes	0.863	0.035	86.3291	0.0452	0.8281	0.864	0.863	0.863	0.828	0.981	0.934
Logistic regression	0.595	0.105	59.4937	0.1343	0.4884	0.596	0.595	0.594	0.492	0.862	0.664
Multilayer perceptron	0.894	0.027	89.3671	0.0429	0.8658	0.894	0.894	0.893	0.867	0.987	0.951
Support vector machine	0.889	0.029	88.8608	0.2257	0.8597	0.889	0.889	0.888	0.860	0.963	0.845
*k*-Nearest neighbours	0.808	0.049	80.7595	0.0677	0.7579	0.811	0.808	0.808	0.759	0.872	0.695
Decision tree	0.646	0.096	64.557	0.1414	0.5488	0.653	0.646	0.646	0.557	0.845	0.603
Random rorest	0.858	0.046	85.8228	0.134	0.8191	0.865	0.858	0.852	0.821	0.985	0.941

TP, true positive; FP, false positive; MCC, Matthews correlation coefficient; ROC, relative operating characteristic; PRC precision–recall curve

**Table 2:** Comparative overview of seven prediction algorithms according to the 81 downregulated genes

	TP rate	FP rate	Accuracy %	Mean absolute error	Kappa	Precision	Recall	*F*-measure	MCC	ROC area	PRC area
Naive bayes	0.846	0.037	84.557	0.0515	0.8055	0.848	0.846	0.846	0.809	0.980	0.926
Logistic regression	0.668	0.082	66.8354	0.1091	0.5828	0.672	0.668	0.669	0.587	0.919	0.758
Multilayer perceptron	0.886	0.029	88.6076	0.047	0.8563	0.885	0.886	0.886	0.858	0.988	0.953
Support vector machine	0.861	0.036	86.0759	0.2264	0.8241	0.861	0.861	0.860	0.826	0.958	0.808
*k*-Nearest neighbours	0.744	0.066	74.4304	0.0884	0.6777	0.744	0.744	0.740	0.677	0.836	0.615
Decision tree	0.618	0.098	61.7722	0.1542	0.5131	0.612	0.618	0.610	0.521	0.847	0.582
Random forest	0.825	0.053	82.5316	0.1386	0.7755	0.827	0.825	0.813	0.781	0.979	0.919

TP, true positive; FP, false positive; MCC, Matthews correlation coefficient; ROC, relative operating characteristic; PRC, precision–recall curve

### TNBC-subtype network analysis

It is of great interest to look for genetic interactions within the few TNBC subgroup signature genes. This can lead to a better understanding of the TNBC-subtype-specific phenotypes than by just considering single gene effects. To identify complex pathways that control essential functions in TNBC-subtype-specific cancerogenesis, we analysed gene networks using the *shortest paths* function of the Metacore analysis suite, allowing for maximum two steps (one extra element as intermediary) to connect the genes in the path. We found interactions between each subtype-specific gene (or its product) and other entities such as binding proteins, enzymes, transcription factors, protein kinases and receptors with enzyme activity, through different regulation mechanisms.

**Fig. 2:**
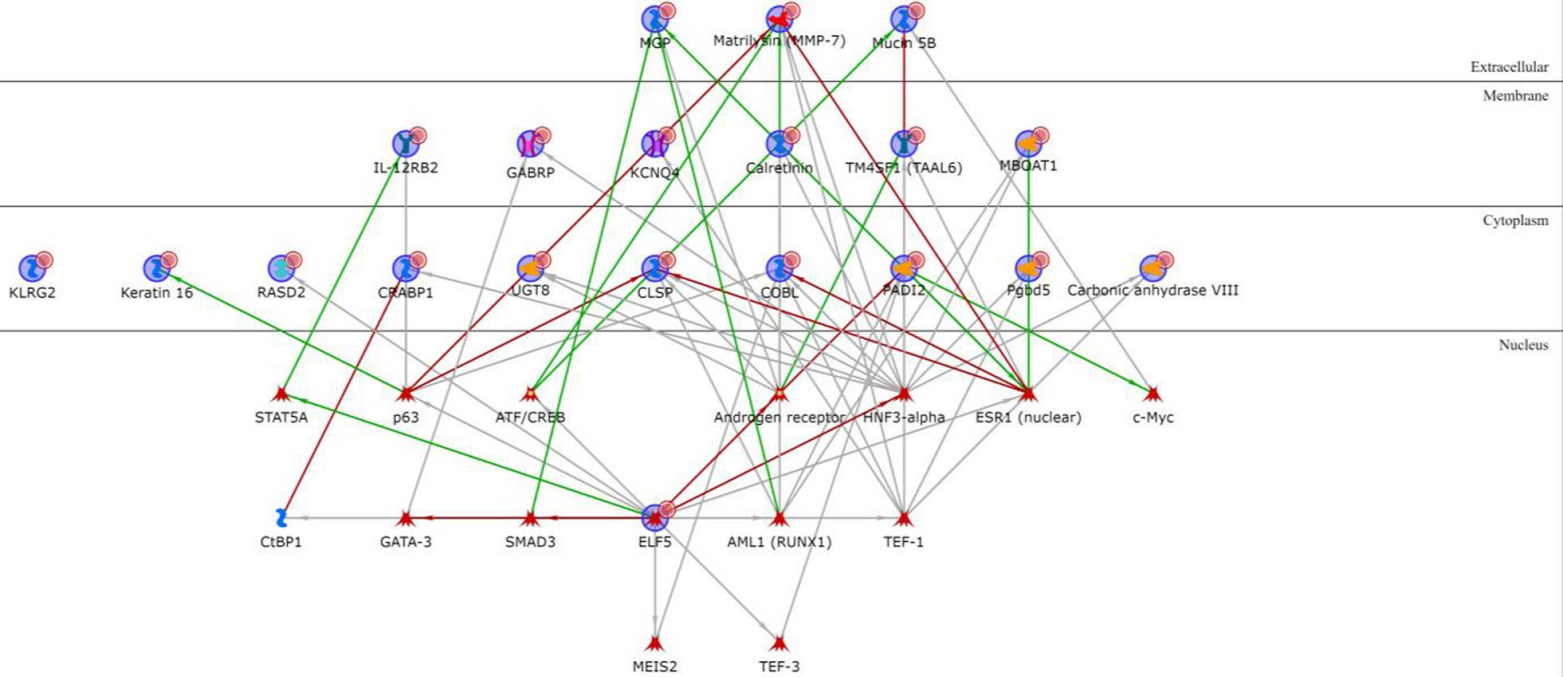
BL1 upregulated genes network analysis. Red arrows refer to inhibition, green arrows to activation and grey ones to unspecified effects, while red circles refer to uploaded differentially expressed genes

**Fig. 3:**
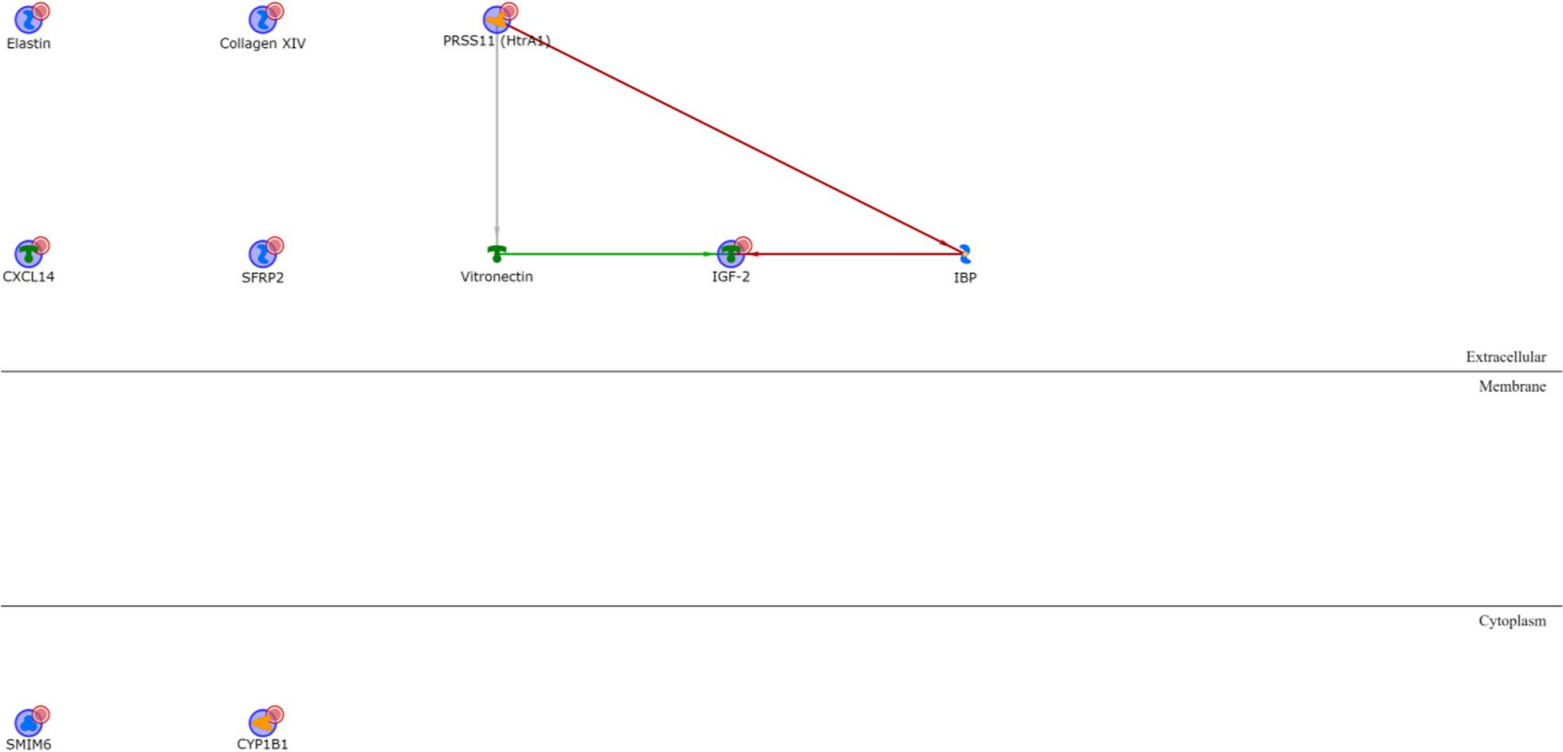
BL1 downregulated genes network analysis

All the BL1 upregulated genes except KLRG2 are connected via one or two transcription factors (Additional file 1: Table S3), with ELF5, PADI2, Matrilysin (MMP7), COBL and CLSP being the most interconnected signature genes and HNF3-alpha, androgen and oestrogen receptors being the most interconnected intermediary transcription factors (Fig. 2). Among the BL1 downregulated genes (Additional file 1: Table S4), only IGF-2 and PRSS11 (HtrA1) are connected via Vitronectin or IBP and the location of all the four proteins is extracellular (Fig. 3).

**Fig. 4:**
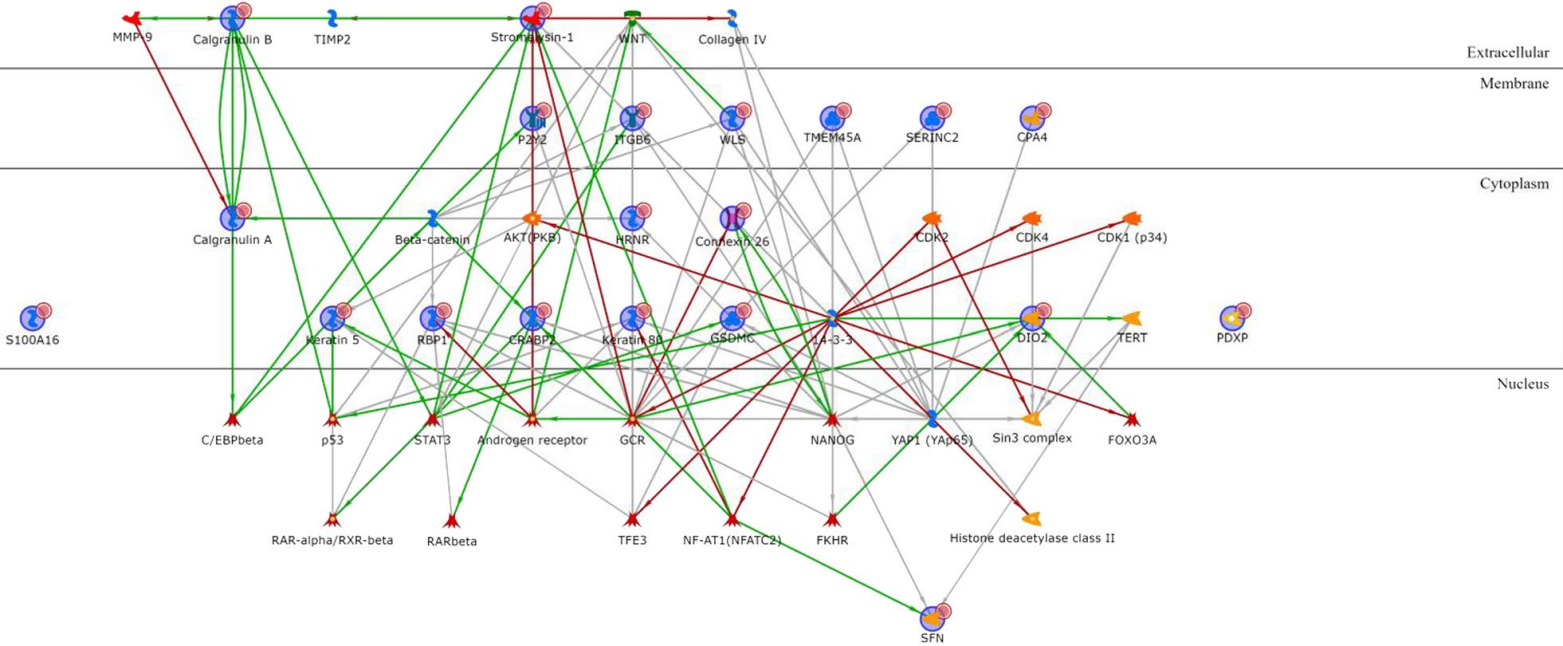
BL2 upregulated genes network analysis

**Fig. 5:**
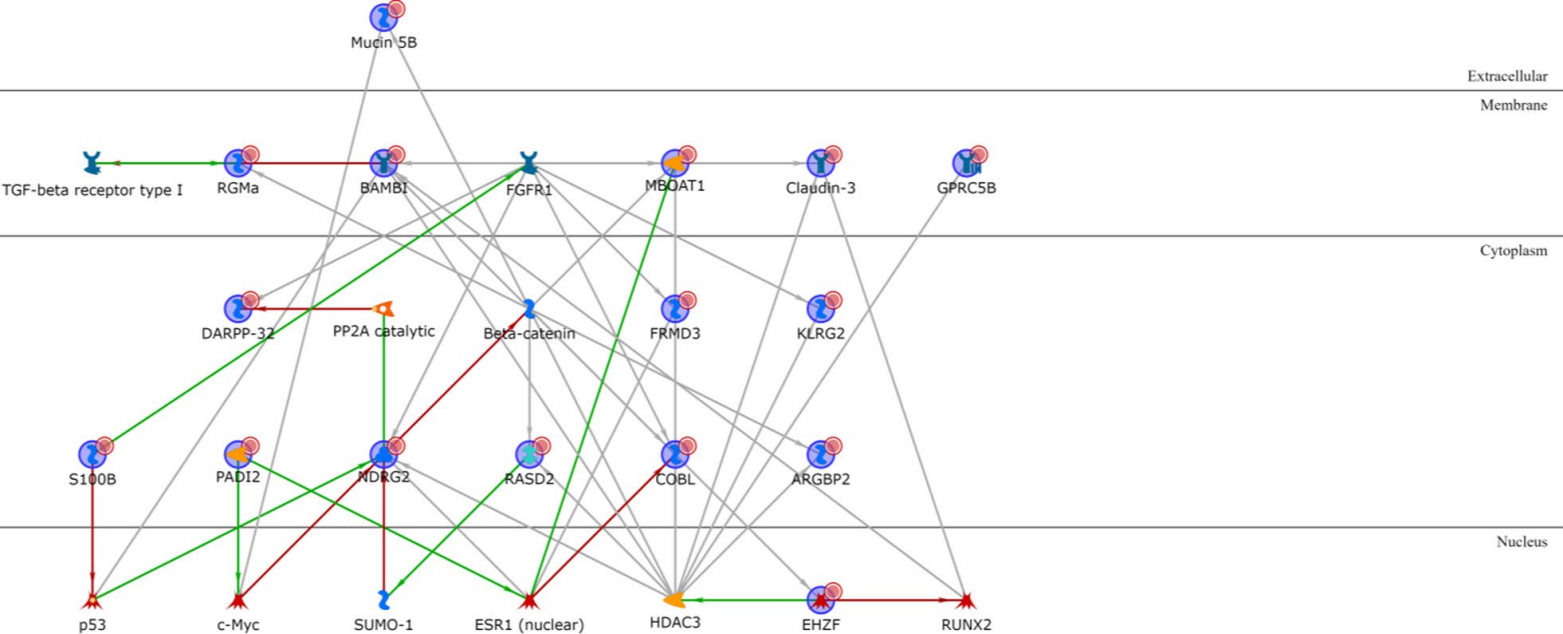
BL2 downregulated genes network analysis

Concerning BL2 upregulated genes (Additional file 1: Table S5), most of them encode for cytoplasmic proteins transcriptionally regulated by a few intermediary transcription factors (p53, STAT3, RAR-alpha, androgen receptor, and FKHR), except for cytoplasmic Calgranulin A that is directly linked to extracellular Calgranulin B via an autoregulatory loop (mutual activation by binding). S100-A16 is not connected to any other upregulated gene, while the only other extracellular product, Stromelysin-1, is transcriptionally regulated by several intermediary transcription factors and is also a therapeutic drug–target (see chapter below). The only nuclear product is SFN, and there are six membrane proteins, all controlled by a few intermediary transcriptional factors (Fig. 4). Among the BL2 downregulated genes (Additional file 1: Table S6), the most interconnected proteins are NDRG2 and COBL, both cytoplasmic, BAMBI and MBOAT1, both located on the cell membrane, and EHZF that is located in the nucleus (Fig. 5).

**Fig. 6:**
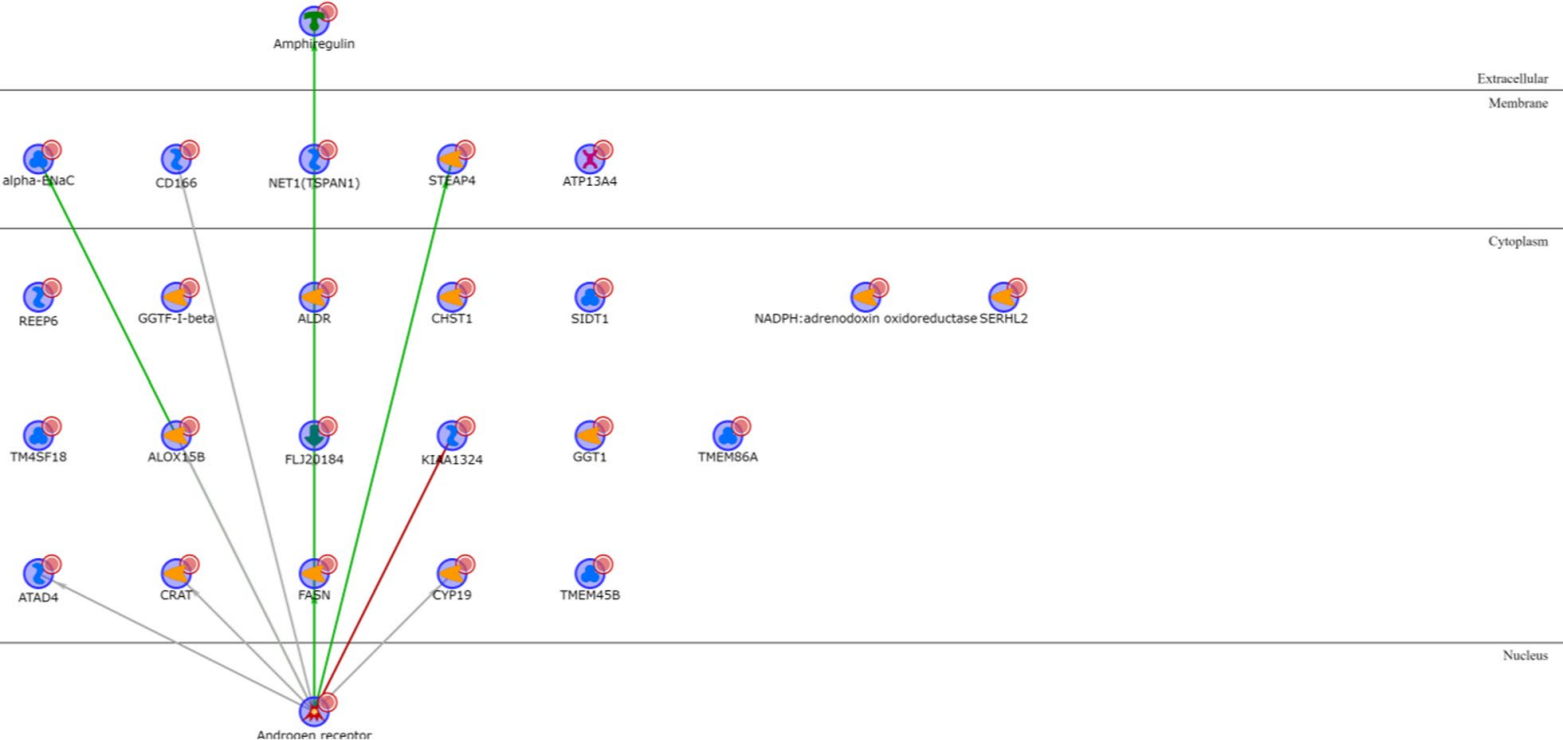
LAR upregulated genes network analysis

**Fig. 7:**
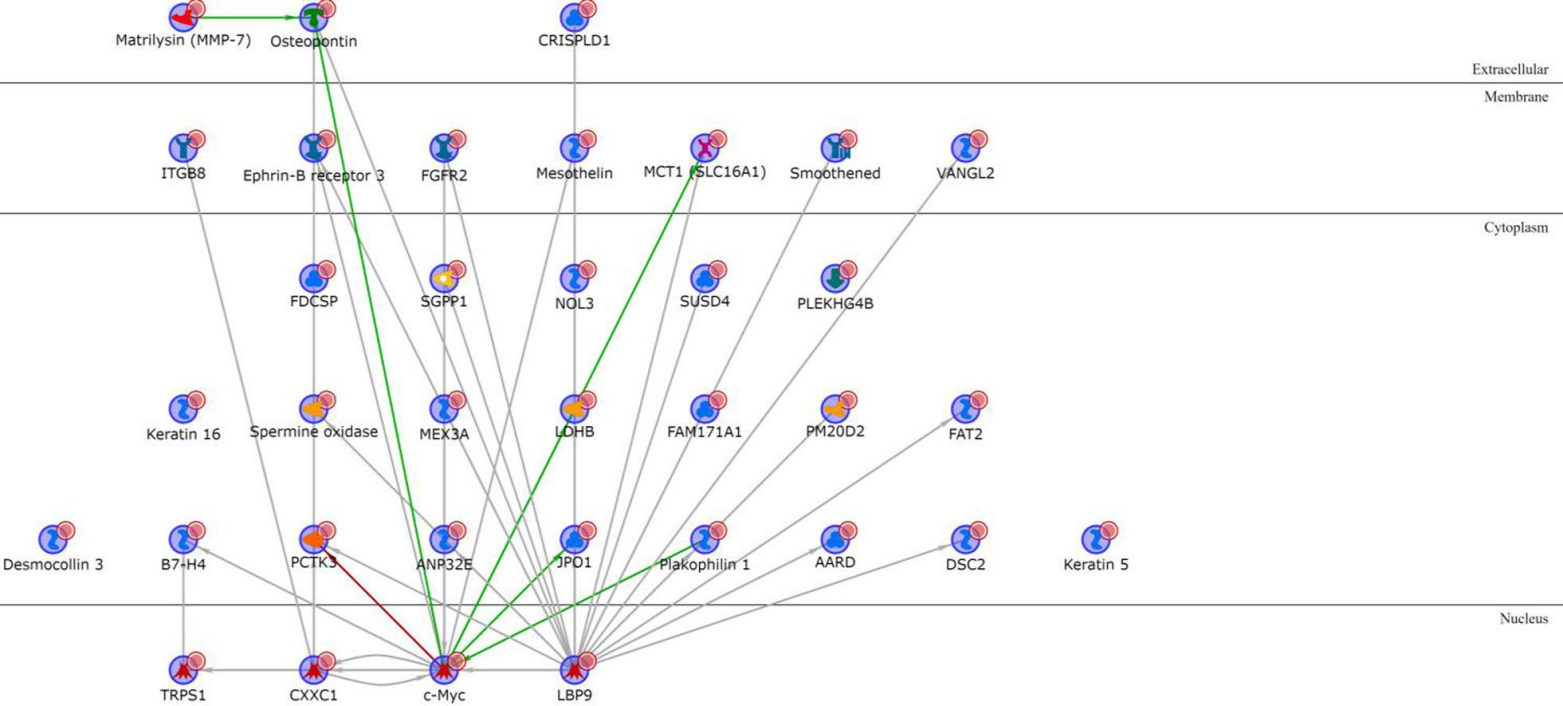
LAR downregulated genes network analysis

Twelve out of the twenty LAR upregulated gene products are directly regulated by the androgen receptor, that is in the LAR signature itself (Additional file 1: Table S7). These include the Amphiregulin extracellular protein; four membrane proteins (alpha-ENaC, CD166, TSPAN1 and STEAP4); and seven cytoplasmic proteins (ALOX15B, FLJ20184, KIAA1324, ATAD4, CRAT, FASN and CYP19) (Fig. 6). Thirty-one out of 35 proteins encoded by the LAR downregulated genes are directly connected without any intermediary (Additional file 1: Table S8), with the transcription factors LBP9, c-Myc and CXXC1 controlling most of the signature genes (Fig. 7).

**Fig. 8:**
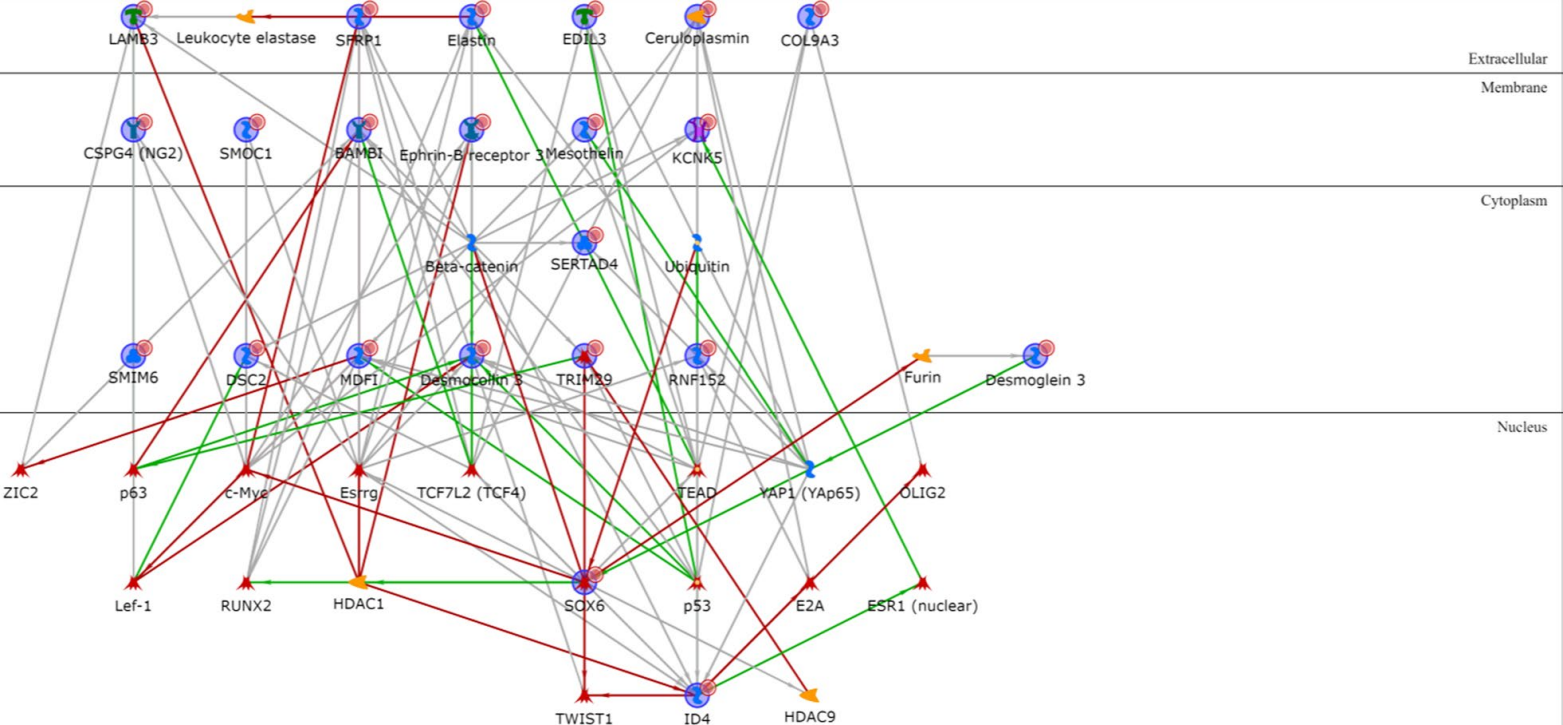
M upregulated genes network analysis

**Fig. 9:**
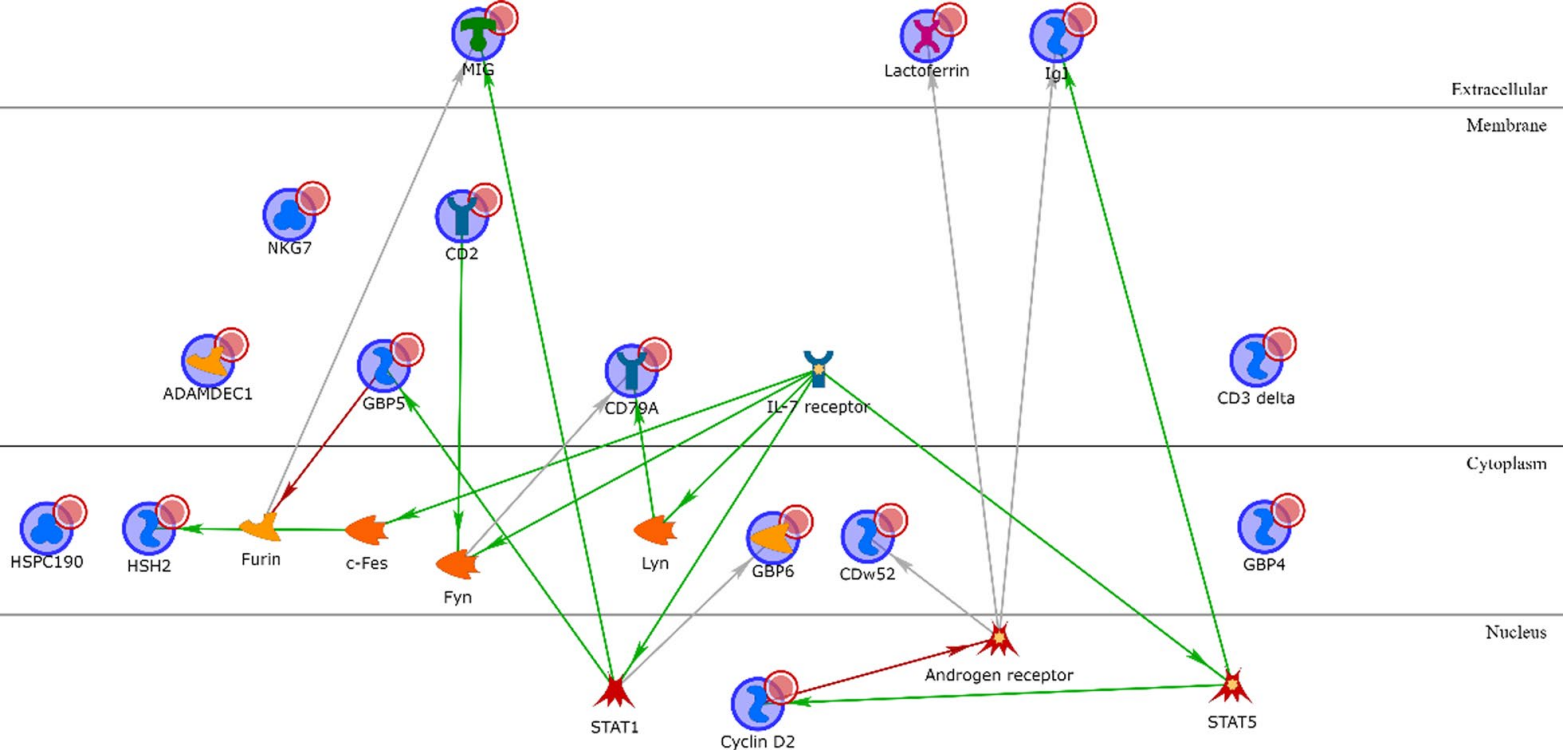
M downregulated genes network analysis

None of the proteins encoded by the M subtype upregulated genes are directly connected with any of the others (Additional file 1: Table S9), but they are all connected if one intermediary is added, with SOX6 and ID4 (nuclear), MDFI and Desmocollin 3 (cytoplasmic), and the BAMBI transmembrane glycoprotein being the most interconnected network hubs (Fig. 8). The network involving the proteins encoded by the downregulated M genes (Additional file 1: Table S10) is not easily interpretable (Fig. 9).

**Fig. 10:**
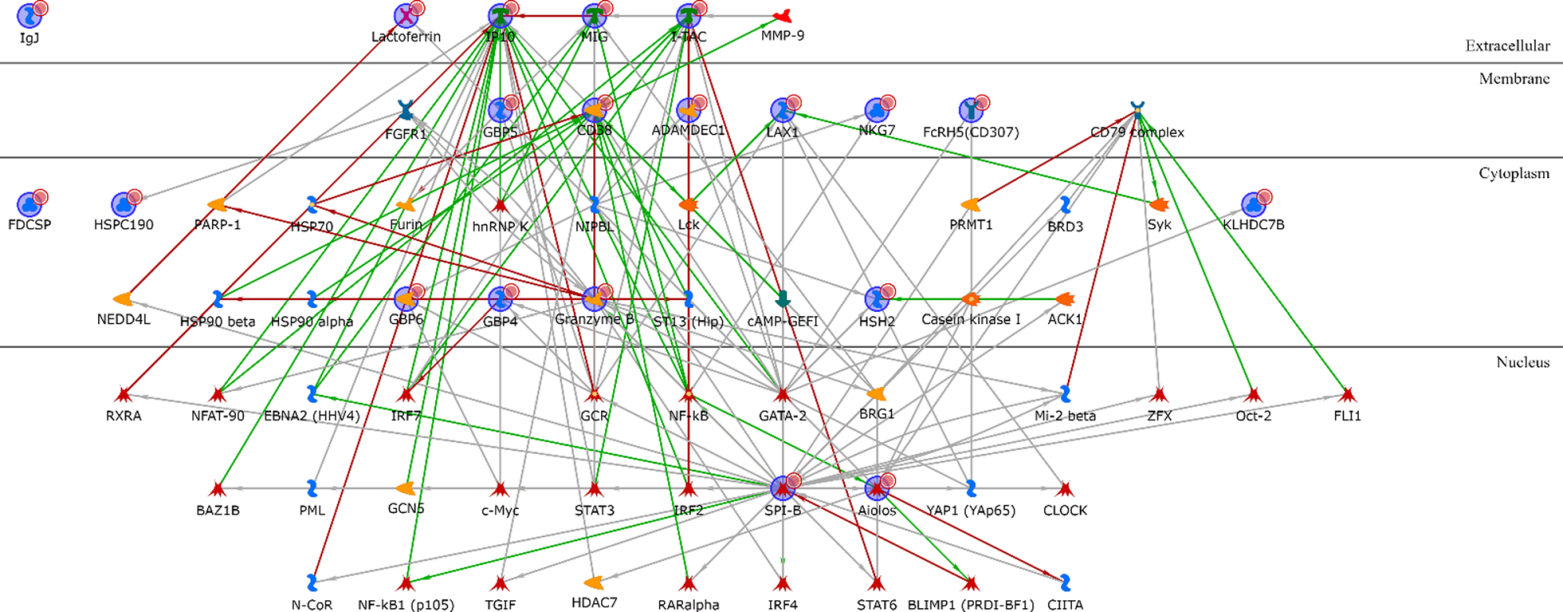
IM upregulated genes network analysis

**Fig. 11:**
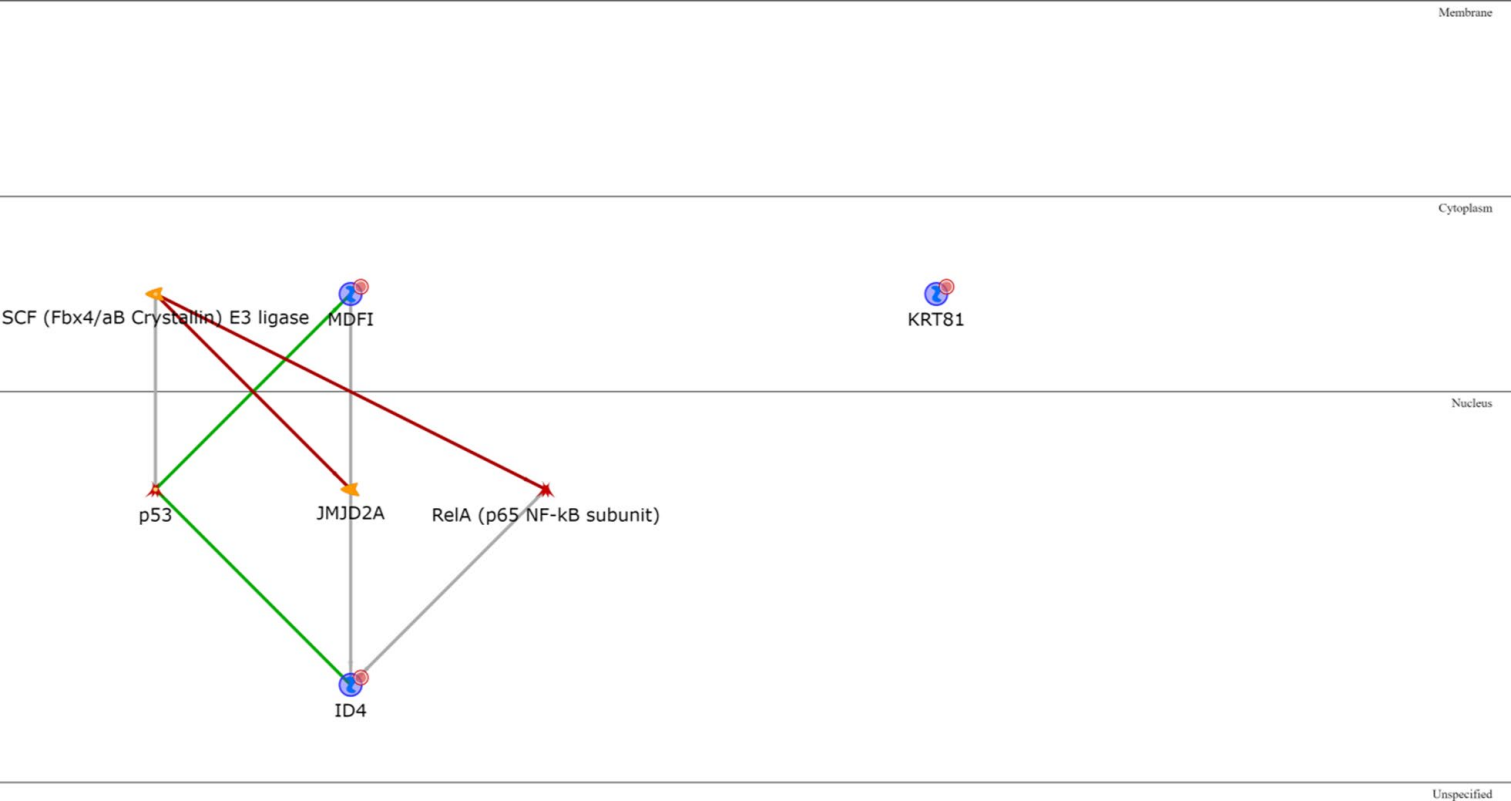
IM downregulated genes network analysis

As for the IM subtype, the only two upregulated genes encode for two transcription factors (Additional file 1: Table S11), SPI-B and Aiolos, that are among the most interconnected within the network when one intermediary is included. The majority of intermediaries converge towards IP-10, MIG or I-TAC, three extracellular chemokines, or to CD38, a type II transmembrane glycoprotein, all overexpressed in the IM subtype. Another central node of the IM network is Granzyme B, a protease secreted by natural killer cells and cytotoxic T lymphocytes (Fig. 10). The IM downregulated genes (Additional file 1: Table S12) are ID4, MDFI and KRT81. Only the proteins encoded by the first two are connected, via either the transcription factor p53 or the demethylase JMJD2A (Fig. 11).

**Fig. 12:**
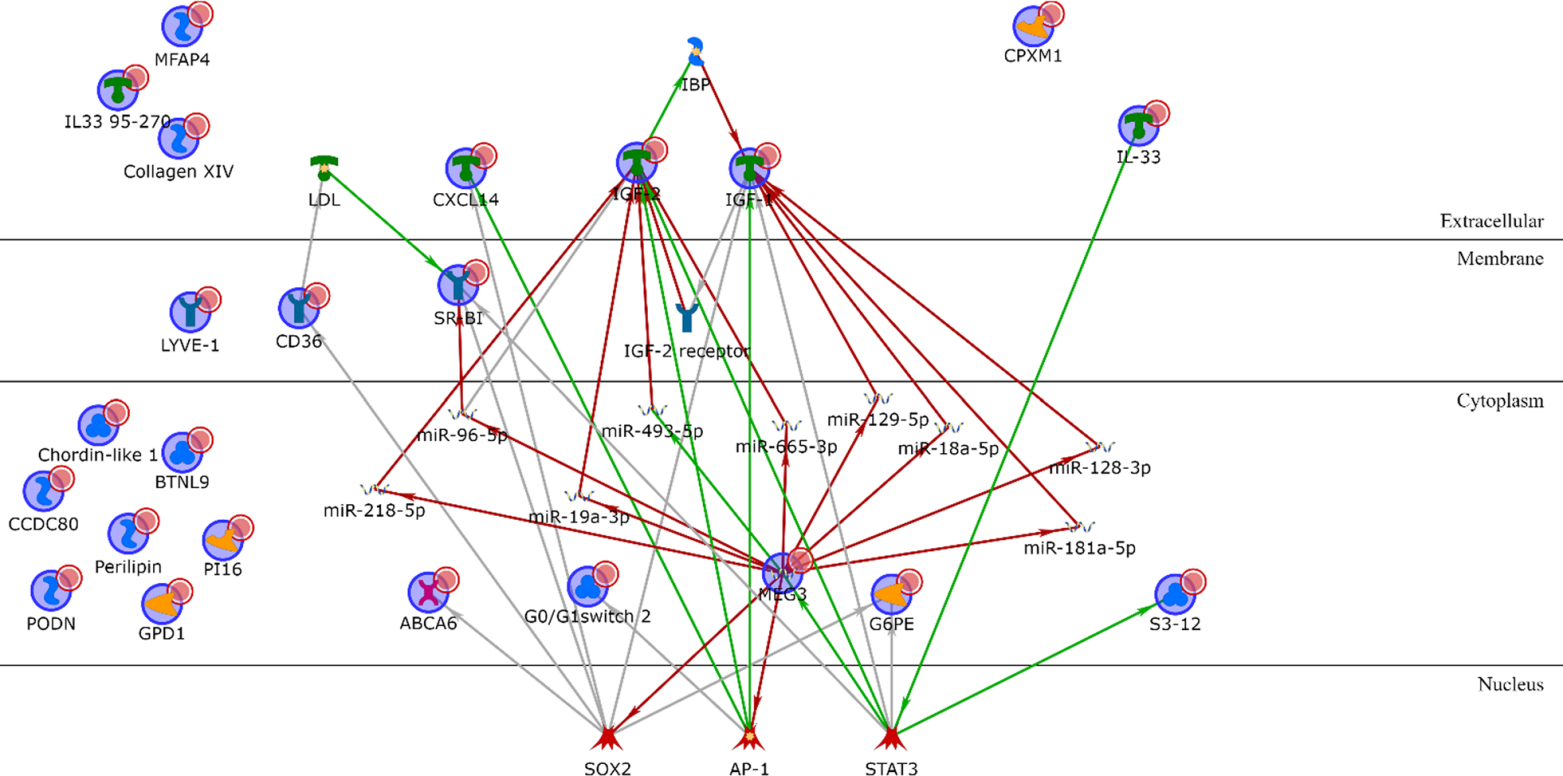
MSL upregulated genes network analysis

**Fig. 13:**
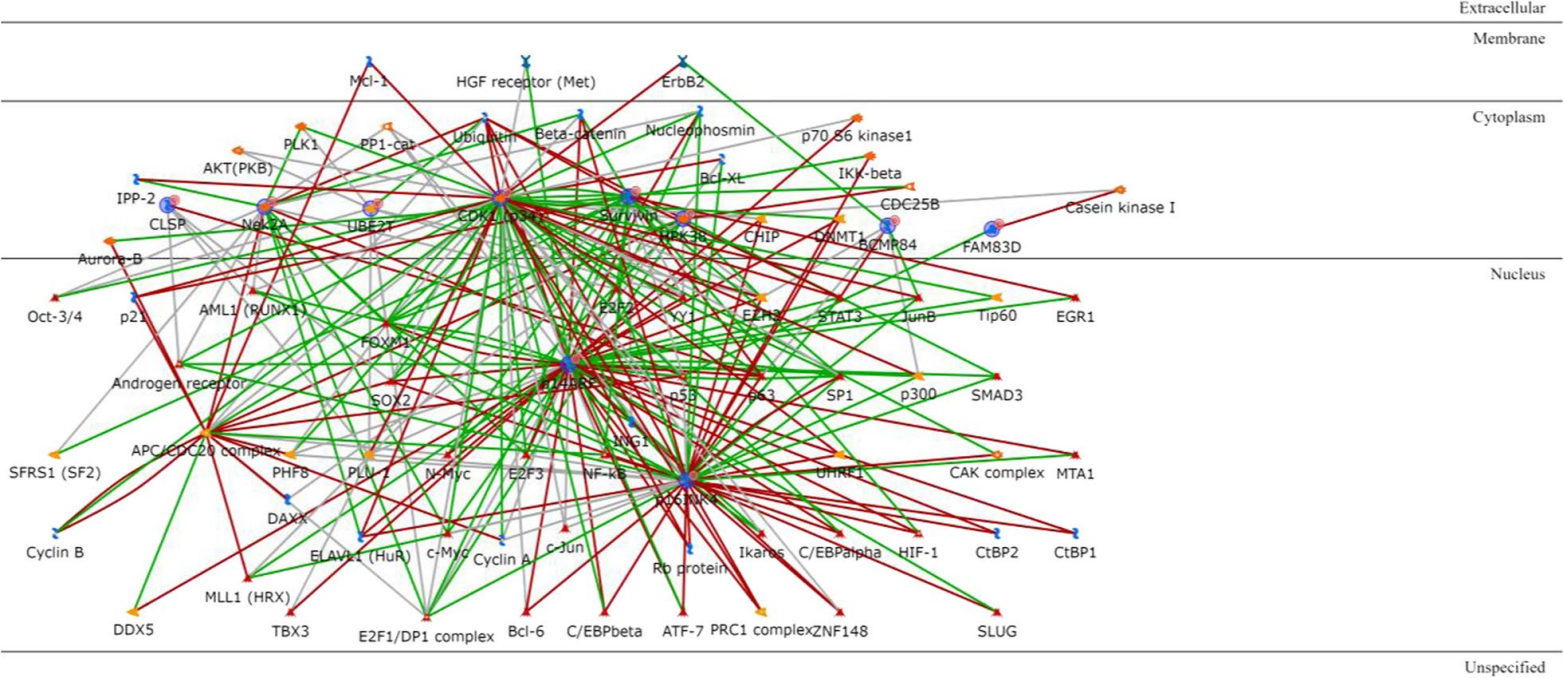
MSL downregulated genes network analysis

Finally, the non-coding gene MEG3 is the central element in the network resulting from the MSL upregulated genes (Additional file 1: Table S13) and is linked to IGF-I and IGF-II via inhibition of several microRNAs (miR-218-3p, miR-96-5p, miR-19-3p, miR-493-5p, miR-665-3p, miR-129-5p, miR-18a-5p, miR-129-3p and miR-181a-5p) targeting the two extracellular growth factors (Fig. 12). On the other hand, cell cycle controlling elements such as CDK1 and CDKN2A (Additional file 1: Table S14) have a central role within the MSL downregulated genes (Fig. 13).

### Identification of druggable targets

The genes differentially expressed in each subtype were subsequently analysed with Metacore, to look for any druggable target.

The most overexpressed BL1 druggable target is Matrilysin, encoded by MMP7 and targeted by several therapeutic inhibitor drugs, such as Batimastat, Marimastat and Rebimastat (Additional file 1: Table S15).

As for the BL2 subgroup, the main therapeutic drug–target inhibitory interaction concerns Stromelysin-1 encoded by MMP3 and targeted by Doxycycline and Tanomastat (Additional file 1: Table S16).

On the other hand, one of the most recurrent and potentially important upregulated LAR druggable targets is androgen receptor encoded by AR and inhibited by Bicalutamide, Diethylstilbestrol, Drospirenone, Finasteride, Flutamide, Metandienone, RU58841, Silibinin and Zanoterone. The second is CYP19 encoded by CYP19A1 and targeted by several aromatase inhibitors, such as Aminoglutethimide, Anastrozole, Exemestane, Letrozole and Testolactone, and then, GGT1, targeted by Acivicin and by Oxiglutathione; GGTF-I-beta, encoded by PGGT1B and targeted by L-778,123; ALDR, encoded by AKR1B1 and targeted by Tolrestat; alpha-ENaC, encoded SCNN1A and targeted by Amiloride (Additional file 1: Table S17). As for the M, IM and MSL subtypes (Additional file 1: Tables S18, S19, S20), no specific therapeutic drug–target interaction was spotted. Conversely, several inhibition secondary drug–targets interactions for the upregulated genes, predicted based on similarities in the structures, were found. Ephrin-B receptor 3, encoded by EPHB3 and upregulated in the M subgroup, is a predicted target of several inhibitory drugs such as CC-223, Dovitinib, Nazartinib, Nilotinib and Ponatinib; CD38 in the IM subgroup is a predicted target of Ca('2+), Fluticasone propionate and Quercetin; SR-B encoded by SCARB1 and overexpressed in the LAR group is a predicted target of beta-cyclodextrin, docosahexaenoic acid and ITX-5061.

Reciprocally, no activating therapeutic drug–target interaction for the downregulated genes was spotted in all the six TNBC subgroups (Additional file 1: Table S21 to Table S26).

### TNBC-subtype prediction

It is very important in any biological study to identify the most meaningful information from complex biological data. It is known that physiological and pathological changes in the tumour phenotype and its sensitivity to specific treatments are generally driven by molecular interactions. Hence, we evaluated if the subtype-specific gene signatures previously described were also able to predict sample classes.

Accordingly, seven different prediction models were applied on the GEO-TN data set, starting from the lists of upregulated (Additional file 1: Table S1) and downregulated (Additional file 1: Table S2) genes previously obtained. For both lists, ten fold cross-validation was used as it gives the models the opportunity to train on multiple train–test splits, giving a better indication of how well the models perform on unseen data. The variable to predict was “TNBC subtype”, and the explanatory features were the up- and downregulated genes.

Tables 1 and 2 summarise the weighted averages across the six classes of the metrics used to judge each model’s performance in classifying the samples using the up- and the downregulated genes, respectively.

The multilayer perceptron (MP), followed by support vector machine (SVM) model, stands out with the best metrics scores; on the other hand, logistic regression (LR) and decision tree (DT) seem to be the least performant among all models, for both lists. Therefore, MP was then picked for further use in external validation on the TCGA-TN and Italian-TN data sets.

Consequently, in order to know if any of the genes had a low predictive weight according to the best predictive model (MP), seven different attribute selection methods were elaborated, which yielded slightly different gene rankings. The genes that were voted by the majority of algorithms as unimportant were then removed (Additional file 1: Table S27).

Following the two gene lists refinement, a per-subgroup ROC comparison was made, before and after attribute selection, to evaluate if the aforementioned gene elimination altered the prediction performance of the same model. The predictions were first measured on the training set with the tenfold cross-validation option and then on the two validation sets. Very stable ROC scores were obtained, even after deletion of the least important genes. In terms of the upregulated genes, despite the removal of 17 genes, the ROC score improved in both the training and the validation data sets, in the majority of cases. The detailed ROC areas by class and the weighted averages are given in Table 3, for upregulated (upper rows) and downregulated genes (lower rows), before and after attribute selection.

**Table 3:** Per-subgroup prediction ROC scores for up- and downregulated genes, before and after attribute selection

	BL1	BL2	M	IM	MSL	LAR	Weighted average	Validation option
	*Per-subgroup prediction ROC metric before attribute selection (Total number of upregulated genes = 120)*							
Upregulated genes	0.979	0.97	0.983	0.992	0.996	0.999	0.987	Cross-validation on GEO set (tenold)
	0.862	0.949	0.837	0.890	0.904	0.784	0.852	Validation set: Italian set
	0.958	0.981	0.958	0.958	0.816	0.92	0.948	Validation set: TCGA set
	*Per-subgroup prediction ROC metric after attribute selection (Total number of upregulated genes = 103)*							
	0.975	0.978	0.984	0.988	0.996	1	0.986	Cross-validation on GEO set (tenfold)
	0.916	0.955	0.843	0.922	0.962	0.7	0.883	Validation set: Italian set
	0.96	0.955	0.974	0.951	0.801	0.864	0.94	Validation set: TCGA set
	*Per-subgroup prediction ROC metric before attribute selection (Total number of downregulated genes = 81)*							
Downregulated genes	0.986	0.977	0.987	0.991	0.988	0.998	0.988	Cross-validation on GEO set (tenfold)
	0.727	0.808	0.924	0.871	0.897	0.886	0.858	Validation set: Italian set
	0.678	0.788	0.861	0.781	0.858	0.985	0.813	Validation set: TCGA set
	*Per-subgroup prediction ROC metric after attribute selection (Total number of downregulated genes = 77)*							
	0.984	0.961	0.984	0.986	0.985	0.996	0.984	Cross-validation on GEO set (tenfold)
	0.742	0.962	0.816	0.815	0.776	0.91	0.83	Validation set: Italian set
	0.743	0.807	0.813	0.776	0.7	0.977	0.802	Validation set: TCGA set

The three validation options are reported

## Discussion

The development of a plausible treatment for TNBC neoplasms is largely hindered by the high heterogeneity of their different phenotypes. Indeed, TNBC patients are pathologically defined by the triple-negative expression of ER, PgR and HER2 receptors and not positively via specific markers that may represent druggable targets.

In this research study, starting from a large data set of TNBC records and applying the classification proposed by Lehman and collaborators, which relies on whole transcriptomic profiles, we were able to define two small-size classifiers, one based on the most overexpressed and the other on the most under-expressed genes within each of the six TNBC subtypes. The models were tested on two independent data sets, in order to evaluate the accuracy of the subtype prediction. The least important genes were discarded, to define a minimum number of genes associated with TNBC subtyping. The final classifiers consisted in 103 upregulated or 77 downregulated genes, most of which had been previously found by several authors to be associated with TNBC or to basal-type BC or to BC in general. Therefore, our results add new important pieces of information that may help clinicians in the classification of TNBC. Knowing that a “one-size-fits-all” treatment approach is questionable for TNBC, molecular subtyping is crucial in determining the best therapeutic option for each single patient.

Concerning the basal-like phenotype, stratified into two further subtypes, we found that genes overexpressed in BL1 tumours are enriched in the major mechanisms that define this particular subtype: cell proliferation and DNA damage response. Most of these genes have been previously associated with the basal phenotype, and our study highlights their BL1-specificity. Specifically, CRABP1, which proved to be under-expressed in hormone-dependent tumours but maintained at high expression levels in triple-negative tumours, inhibits retinoic acid which should normally inhibit growth and induce apoptosis [17]. GABRP was already proven to be critical for TNBC cell growth [18], and its inhibition was reported to suppress basal-like BC progression [19]. Likewise, Powell et al. reported that the majority of breast carcinomas that stain with CALB2 are more likely to be high-grade, ER-negative and display a basal-like phenotype [20]. TM4SF1, as well, is known to be downregulated in hormone-positive tumours [21], while increased expression of MMP7 distinguishes the basal-like breast cancer subtype from other triple-negative tumours [22, 23]. Indeed, Matrilysin is a validated target of several compounds that could be proposed to personalise BL1 TNBC. At the same time, PGBD5 levels were found significantly higher in basal-like BC [24], and the same goes for CALML5, one of the top expressed genes in TNBC samples [25], PADI2 [26] and KLRG2 [27]. Gong et al. demonstrated that the upregulation of MGP promotes the proliferation of cancer which probably makes it a novel biomarker or therapeutic target for TNBC patients [28]. The same was also reported for KRT16 by Lehmann et al., who showed its differential expression in the basal-like subtype [10], and confirmed by our Metacore analysis that revealed this basal cytokeratin as the predicted target of L-Triiodothyronine. Two other predicted drug targets within the BL1 signature are KCNQ4, targeted by Bepridil and Fampridine, and CA8 encoding carbonic anhydrase VIII and targeted by Foscarnet.

Among the seven downregulated genes in BL1, COL14A1 [29], CYP1B1 [30] and ELN had been previously associated with TNBC. The latter was considered in a TNBC genetic signature [31], in line with our findings. On the other hand, HTRA1 was found to be significantly expressed within the breast normal ductal glands and its expression is significantly downregulated in invasive breast cancer in general [32]. Our study therefore confirms and specifies its down-modulation in the BL1 subtype.

The BL2 subtype is mainly defined by the abnormal over-activation of several signalling pathways such as Wnt/β-catenin; indeed, one of the overexpressed genes found in our study is WNT7B, also reported by several studies in governing BC generally and TNBC more specifically [33]. Through the latter, another BL2 gene (WLS) promotes the proliferation of breast cancer cells [34]. In terms of S100A9/8, Bergenfelz was the first to report that it can be considered as a novel therapeutic target for patients with ER(−) PgR(−) breast cancers [35] followed by several other studies [36]. Indeed, our Metacore analysis identified Calgranulin B, encoded by S100A9, as the predicted target of Paquinimod as well as of Tasquinimod. Gene expression studies have previously identified KRT5 mRNA in normal breast and basal-like breast cancer, and monoclonal antibodies against KRT5 have been used to identify basal-like TNBC [37]. This basal cytokeratin has been identified as a predicted target of Androstanolone by our analysis; however, it is widely expressed in normal gland structures such as salivary and sweat glands and therefore targeting it may be critical. Previous findings indicated that CRABP2 promotes invasion and metastasis of ER^−^ breast cancer. No studies to date have demonstrated the direct involvement with the BL2 phenotype of CPA4 [38], TMEM45A [39], S100A16 [40], COL4A5, GSDMC, MMP3, ITGB6, or GJB2 [41]. However, our drug interaction analysis revealed that GJB2 is a predicted target of beta-Cyclodextrin.

On the other hand, Kloten et al. reported the loss of NDRG2 protein expression in human BC and low NDRG2 immunoreactivity in TNBCs [42], which goes in line with the significant downregulation we found in the BL2 subgroup. SORBS2, another gene downregulated in BL2, is a tumour suppressor that was reported by Alsafadi et al. as a candidate marker to predict metastatic relapse in BC [43]. In terms of PADI2 gene, we found that—as mentioned before—it is significantly highly expressed in BL1 subtype, contrary to BL2 subtype where it is significantly lowly expressed. Therefore, it can be proposed as a potential biomarker for differential diagnosis within the basal-like TNBC tumours. This has also prognostic implications as the BL1 subtype showed a significantly higher response rate to chemotherapy than the BL2 [44, 45].

The mesenchymal-like subtype (M) is mainly defined by a variety of signalling pathways, such as extracellular matrix–receptor interactions and gap junctions, which can explain the differential overexpression of DSG3 compared to the other subtypes [46]. The latter operates by facilitating cancer cell growth and invasion by controlling E-cadherin-Src signalling and cell–cell adhesion. The same goes for COL9A3 [47], which is involved in matrix synthesis and controls its degradation. It was also identified as significantly associated with the prognosis of TNBC in an independent prognostic signature [48]. MSLN has been explored by several studies and found to promote epithelial-to-mesenchymal transition and tumorigenicity [49]. This can explain its overexpression in this particular TNBC phenotype as also reported by Del Bano et al. [50]. ID4 was reported to be highly expressed in TNBCs by Donzelli et al. [51], and it acts as an oncogene. Shen et al. found that the majority of ER-negative breast cancer cells expressed moderate to high levels of KCNK5 protein, whereas minimal/low levels of KCNK5 were detected in ER-positive cells [52]. SOX6 has also been investigated by Mehta et al. who found it had an emerging role in BC development and maintenance as well as an involvement in the mesenchymal phenotype [53]. A set of genes found to have a promoter and primordial role in TNBC-related epithelial-to-mesenchymal transition includes: EPH [54], EDIL3 [55] and TRIM29. On the other hand, no analysis has explored MDFI, CSPG4, CP, LAMB3, RNF152, BAMBI, SERTAD4 and SFRP1 to show their involvement in promoting the mesenchymal phenotype of TNBC, while ILRG is a predicted target of Nedocromil.

As for the immunomodulatory subtype (IM), mainly enriched in immune cell markers and signalling, it turned out that all the genes overexpressed in this subtype, according to our analysis, are involved in the tumour immune infiltrate: CD79A [56], CXCL10 [57] and CXCL9 [58] which proved to be a potential biomarker of immune infiltration associated with favourable prognosis in ER-negative BC; GZMB [59], KLHDC7B [60], LTF [61], GBP5 [62] and CXCL11 [63], which were found to be significantly overexpressed in the plasma of breast cancer patients compared to healthy controls; LAX1, which was reported by Mamoor et al. as associated with survival in TNBC; IKZF3, which contributes to the immunologic phenotype of TNBC [64]. A very recent study showed the prognostic value of tumour-infiltrating B lymphocytes along with CD38 and plasma cells in TNBC [65]. All the remaining genes have been confirmed to be associated with immune-induced pathways along with breast cancer, but not specifically triple negative, thus contributing to a better refinement of TNBC.

Regarding the mesenchymal stem-like subtype (MSL), by definition it expresses low levels of cell proliferation-related genes and high levels of stemness-related genes [66]. This is supported by the genes we found downregulated, such as CDK1, or overexpressed, such as IGF1 [67] and IGF2 [68], as well as CXCL14 [69]. The long non-coding RNA MEG3 is generally downregulated in BC, but it has been found highly expressed in Hs578T TNBC cells [70]. Conversely, ID4 and MDFI are highly expressed in the M subtype but downregulated in the IM subtype. On the other hand, CALML5 is overexpressed in BL1 but downregulated in the MSL subtype. Ehmsen et al. reported that S100A14 is overexpressed in epithelial-like, but not in mesenchymal-like phenotype [71], which converges with our findings.

The LAR subtype, even though it does not express the ER receptor, shows highly activated hormonal-related signalling pathways. Lehman et al. reported that tumours within the LAR group expressed numerous downstream AR targets and coactivators such as ALCAM and FASN [10], which were both contained in our LAR-related signature. We found that six of the upregulated LAR genes, among which AR itself, are experimentally validated druggable targets of up to 30 existing compounds. However, AR targeting in TNBC [72, 73] has not achieved so far the expected efficacy. In an inverse perspective, Bhattarai et al. [74] suggested a new refinement of the classification of TNBC by introducing quadruple-negative BC based on AR expression negativity (Additional file 1).

## Conclusion

Our study took full advantage of available TNBC data sets to stratify samples and genes into distinct subtypes, according to gene expression profiles. The development of a data mining approach to acquire a large amount of information from several data sets has allowed us to identify a well-determined number of genes that may help in the recognition of TNBC subtypes. Although further empirical experiments that can serve as validation for the robustness and relevance of the selected genes are needed, our study identified a small number of genes can be tested in the clinics without the need of whole transcriptomic approaches. Most of the signature genes have been previously found to be associated with (triple negative) breast cancer and/or have the potential to become novel diagnostic markers and/or therapeutic targets for specific TNBC subclasses.

## Potential implications

Overall, our refined genetic signatures for each TNBC subtype may provide a simple clinical tool, affordable by most pathology departments, that might contribute to explore TNBC heterogeneity and identify the appropriate treatment for each patient based on the subtype-specific druggable targets. Novel clinical trials taking into account the molecular portrait of the tumour are in fact under development, for TNBC as well.

## Supplementary Information

**Additional file 1**. **Table 1.** List of the most 120 Up-regulated genes with their respective statistics according to MWU and LIMMA tests. **Table 2.** List of the most 81 down-regulated genes with their respective statistics according to MWU and Limma tests. **Table 3.** Interactions Report of BL1 up-regulated genes. **Table 4.** Interactions report of BL1 down-regulated genes.

## Data Availability

The data set supporting the results of this article is available in Gene Expression Omnibus repository, under the GEO accession number [GSE206912].

## Abbreviations

AR: Androgen receptor
BL: Basal-like
BLIA: Basal-like immune-activated
BLIS: Basal-like immunosuppressed
CNV: Copy number variations
DEGs: Differentially expressed genes
DT: Decision tree
ER: Estrogen receptor
FISH: Fluorescence in situ hybridization
FP: False positive
FUSCC: Fudan University Shanghai Classification System
GDC: Genomic data commons
GEO: Gene expression Omnibus
HER2: Human epidermal growth factor receptor 2
IM: Immuno-modulatory
KNN: *K*-nearest neighbours
LAR: Luminal androgen receptor
LIMMA: LInear models for microarray analysis
lncRNAs: Long-non-coding RNAs
LR: Logistic regression
M: Mesenchymal
MCC: Matthews correlation coefficient
MES: Mesenchymal
MP: Multilayer perceptron
MSL: Mesenchymal stem like
MWU: Mann–Whitney *U*
NB: Naive Bayes
PgR: Progesterone receptor
PRC: Precision-recall curve
RF: Random forest
RNAseq: RNA sequencing
ROC: Receiver operating characteristic
SVM: Support vector machine
TCGA: The cancer genome atlas
TILs: Tumor infiltrating lymphocytes
TN: Triple negative
TNBC: Triple-negative breast cancer
TP: True positive
UNS: Unstable
